# Supplementary material for: Analysis of Human Papilloma Virus Content and Integration in Mucoepidermoid Carcinoma
Source: Viruses. 2022 Oct 26;14(11):2353. doi: 10.3390/v14112353 (PMC9698779; doi:10.3390/v14112353)
Supplement: Supplementary file 1 [file viruses-14-02353-s001.zip › viruses-1984634-supplementary.pdf]

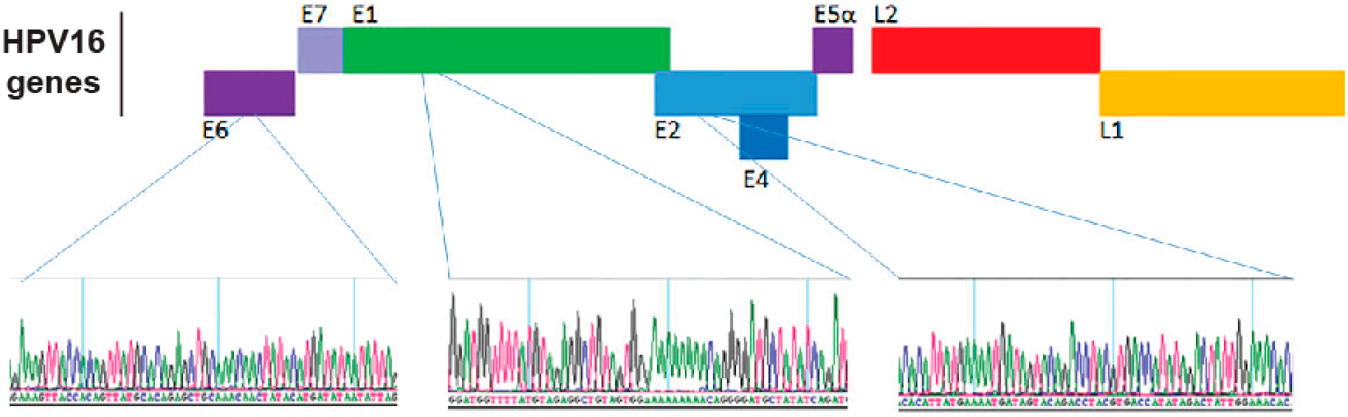

Figure S1. HPV16 PCR and Sanger sequencing analysis. We independently isolated genomic DNA from the FFPE block for MEC1 and performed PCR and Sanger sequencing on three independent regions of the HPV16 genome. Representative fragments of the Sanger traces validating the presence of HPV16 DNA are shown.

Supplemental Table S1. Validation of HPV DNA in tumor MEC1. Relative HPV16 read counts identified by mapping targeted capture DNA sequencing data to a HPV16 reference for the MEC samples that underwent targeted DNA analysis.

MEC_ID	HPV_type	count_of_reads
MEC001	HPV16	1034
MEC003	-	0
MEC006	-	0
MEC007	-	0
MEC008	-	0
MEC009	-	0
MEC010	-	0
MEC011	-	0
MEC012	-	0
MEC013	-	0
MEC015	-	0
MEC016	-	0
MEC017	-	0
MEC018	-	0
MEC019	-	0
MEC020	-	0
MEC021	-	0
MEC022	-	0
MEC023	-	0
MEC024	-	0

MEC025	-	0
MEC027	-	0
MEC028	-	0
MEC033	-	0
MEC035	-	0
MEC036	-	0
MEC038	-	0
MEC039	-	0
MEC040	-	0
MEC041	-	0
MEC045	-	0
MEC046	-	0
MEC079	HPV16	3
MEC084	-	0
MEC087	-	0
MEC089	-	0
MEC096	HPV16	2
MEC098	-	0
MEC114	HPV16	3
MEC117	-	0
MEC121	-	0
MEC123	-	0
MEC136	-	0
MEC160	-	0
MEC167	-	0
MEC170	-	0
MEC173	HPV16	4

Supplemental Table S2. Total mapped reads and mapped rates for MEC1 and MEC23.

Sample	Total Reads	% Mapped	% Uniquely mapped
MEC001	5520841	99.15	87.23
MEC023	13382425	99.94	94.58

Supplemental Table S3. List of targeted sequencing-derived HPV to host integration sites detected in MEC1 using SearcHPV.

Num	Pos	SP	PE	Conf	HPVPos	HumGene	HPVGene
1	chr1:211823777	1	5	low	3308	RP11-354K1.1	Intergenic
2	chr10:15505686	2	4	low	85	Intergenic	E1
3	chr10:69656519	1	7	low	1254	SIRT1	E1
4	chr10:98436051	3	3	high	3094	PIK3AP1	E5_ALPHA

5	chr13:53617505	2	4	low	76	OLFM4	E1
6	chr14:28489344	2	4	low	4865	Intergenic	L1
7	chr17:14276448	4	4	high	76	Intergenic	E1
8	chr19:16564680	6	6	high	1252	EPS15L1	E1
9	chr2:135457871	4	5	high	1241	TMEM163	E1
10	chr20:22781842	2	4	low	85	Intergenic	E1
11	chr21:39093507	2	4	low	7510	KCNJ6	E6
12	chr3:82745849	2	4	low	345	Intergenic	E1
13	chr4:121898294	2	4	low	1293	Intergenic	E1
14	chr4:157323326	3	6	high	3087	Intergenic	E5_ALPHA
15	chr4:36141816	6	6	high	6518	ARAP2	URR
16	chr5:15126295	3	5	high	93	Intergenic	E1
17	chr5:6550306	2	4	low	7308	Intergenic	E6
18	chr5:87613860	5	5	high	812	TMEM161B-AS1	E1
19	chr6:131191962	2	4	low	1254	EPB41L2	E1
20	chr7:75277666	2	4	low	3864	HIP1	L2
21	chr8:56142436	2	4	low	76	XKR4	E1
22	chrX:138890126	2	4	low	76	ATP11C	E1
